# Genomic Diversity of Aurochs From a Mediterranean Ice‐Age Refugium

**DOI:** 10.1111/mec.70449

**Published:** 2026-06-29

**Authors:** Vlatka Cubric‐Curik, Dinko Novosel, Zvonimir Mijadžiković, Ivana Keršić, Maja Ferenčaković, Siniša Radović, Aurélien Capitan, Mekki Boussaha, Jesper Stenderup, Ana Prohaska, Eske Willerslev, Ino Curik, Preston T. Miracle

**Affiliations:** ^1^ Department of Animal Science University of Zagreb Faculty of Agriculture Zagreb Croatia; ^2^ Croatian Veterinary Institute Zagreb Croatia; ^3^ Institute for Quaternary Palaeontology and Geology Croatian Academy of Sciences and Arts Zagreb Croatia; ^4^ Université Paris‐Saclay INRAE, AgroParisTech, GABI Jouy‐en‐Josas France; ^5^ Section for GeoGenetics, Globe Institute University of Copenhagen Copenhagen Denmark; ^6^ Ancient Environmental Genomics Initiative for Sustainability, Globe Institute University of Copenhagen Copenhagen Denmark; ^7^ Department of Genetics University of Cambridge Cambridge UK; ^8^ Centre for Ancient Environmental Genomics, Globe Institute University of Copenhagen Copenhagen Denmark; ^9^ Hungarian University of Agriculture and Life Sciences (MATE), Institute of Animal Sciences Kaposvár Hungary; ^10^ McDonald Institute for Archaeological Research University of Cambridge Cambridge UK

**Keywords:** aDNA, aurochs, genomic diversity, Mediterranean, Pleistocene refugia

## Abstract

The aurochs, the wild ancestor of domestic cattle, was a keystone herbivore in Late Pleistocene Eurasian ecosystems and a major prey species for Palaeolithic hunter‐gatherers. Despite its significance, the genetic structure of aurochs populations that survived the Last Glacial Maximum (LGM) remains poorly understood, especially in southeastern Europe. Here, we present the first directly dated ancient genomes of aurochs from the eastern Adriatic region, recovered from the Upper Palaeolithic site of Šandalja (Istria, Croatia). Two female individuals, dated to approximately 14,800–14,200 and 11,800–11,400 calibrated years before present, were sequenced for low‐coverage whole genomes and near‐complete mitochondrial genomes. Bayesian phylogenetic analyses and median‐joining network reconstruction place both specimens within mitochondrial haplogroup P, the dominant European aurochs lineage. However, they do not cluster within the main P sub‐haplogroup observed in most ancient aurochs samples and in modern cattle carrying P‐type mitochondrial lineages. Instead, one specimen is placed within an ‘alternative’ P sub‐haplogroup, whereas the position of the other appears more isolated and should be interpreted cautiously, as it may be influenced by limited sequence coverage and the resulting uncertainty in phylogenetic placement. At the nuclear genomic level, the two Šandalja aurochs show affinity to Late Pleistocene and Early Holocene aurochs from Italy. Although based on a limited number of specimens, this pattern is consistent with possible genetic connectivity across the Adriatic region, potentially associated with the now‐submerged Great Adriatic Plain (GAP). Overall, our results suggest regional structure among Late Pleistocene aurochs, potentially associated with the exposed Adriatic Plain as a refugium and dispersal corridor between the Apennine and Balkan Peninsulas. By filling a major geographic and temporal gap in the aurochs genomic record, this study highlights the Adriatic Basin as a potentially overlooked centre of Pleistocene megafaunal diversity and refines models of postglacial recolonization and cattle evolutionary history.

## 1 Introduction

The aurochs ( *Bos primigenius* ) was one of the most important large herbivores of the Late Pleistocene, exerting a strong influence on the structure of European and Eurasian ecosystems; it was also common prey of Eurasian Palaeolithic hunter‐gatherers (Gamble 1986; Klein 2009; Mellars 1996; Miracle 2007a; Yeshurun et al. 2025). As the ancestor of modern domestic cattle ( *Bos taurus* ), it is a key species for understanding cattle evolution and domestication. Aurochs were first domesticated in the Fertile Crescent about 11,000 years ago (Ajmone‐Marsan et al. 2010; Clutton‐Brock 1999; Rossi et al. 2024). During the coldest phases of the Pleistocene, aurochs are not found in northern Europe (Wright 2016), and most likely retreated into several major glacial refugia where climatic conditions remained sufficiently mild to allow long‐term survival. These isolated populations formed the basis for later postglacial expansions and the recolonization of Europe during the Holocene. Understanding their genetic structure is therefore essential for reconstructing the phylogeographic patterns and evolutionary processes that shaped subsequent aurochs populations and for interpreting the deep evolutionary roots of genetic diversity observed in domestic cattle today.

**FIGURE 1:**
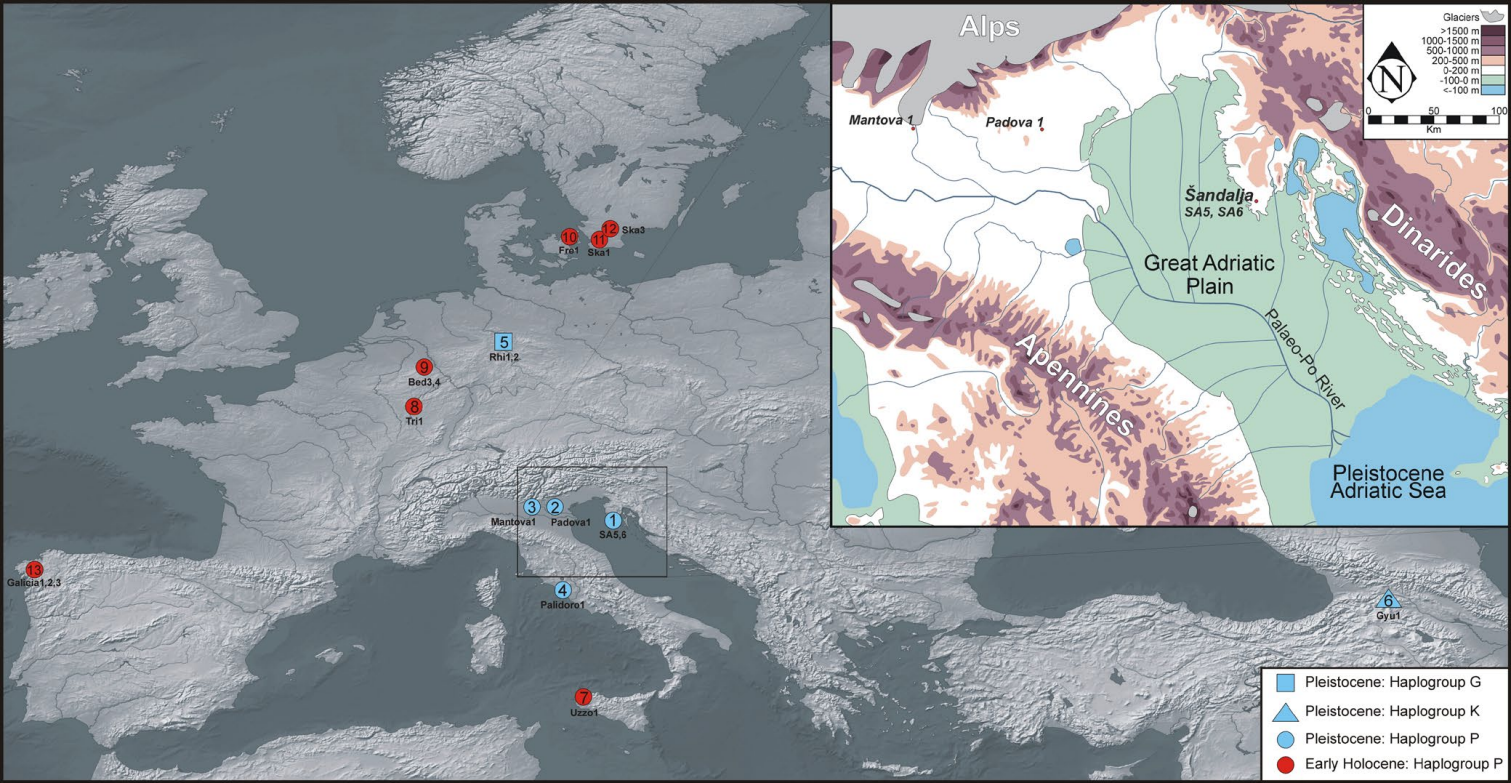
Location of Šandalja and other directly ^14^C‐dated aurochs sequences from the Pleistocene (> 11,700 calBP) and early Holocene (11,700–9,000 calBP) periods. (1) HRV_SA5_11612, HRV_SA6_14757; (2) ITA_Padova1_14005; (3) ITA_Mantova1_13052; (4) ITA_Palidoro1_18017; (5) DEU_Rhi1_51080, DEU_Rhi2_46087; (6) ARM_Gyu1_13906; (7) ITA_Uzzo1_9486; (8) DEU_Tri1_10060; (9) DEU_Bed3_11564; DEU_Bed4_11639; (10) DNK_Fre1_10587; (11) SWE_Ska1_9335; (12) SWE_Ska3_9546; (13) ESP_Galicia1_9250, ESP_Galicia2_9217, ESP_Galicia3_9257. The base map of Europe was obtained from the Natural Earth public domain map dataset (https://www.naturalearthdata.com/downloads/10m‐raster‐data/10m‐gray‐earth/). The inset map of the northern Adriatic was drawn by Miracle with late‐glacial Adriatic Sea reconstructed for MIS2 based on Miracle (1995, 2007b) and glacier extent, river courses and lakes on the Adriatic Plain, based on Falcucci and Peresani (2021) and Žebre et al. (2021).

During the Last Glacial Maximum (LGM), approximately 26,000–19,000 years ago, a dramatic sea‐level drop of 120–130 m (Lambeck et al. 2004, 2014) exposed large portions of the northern Adriatic Basin (Dean et al. 2020; Forenbaher 2020; Miracle 1995, 2007b; Mussi 2001). The area now covered by the sea became an extensive landmass—the Great Adriatic Plain (Figure 1), which connected Istria and the Dinaric hinterland with the Po River Valley on the Apennine Peninsula (Maselli et al. 2014; Miracle 2007b; Mussi 2001). This vast Adriatic lowland was predominantly a steppe and grassland ecosystem rich in resources, including abundant freshwater sources and floodplain zones, which supported the survival and migration of numerous megafaunal species. Faunal remains from the Upper Pleistocene in the Istrian region confirm that this palaeolandscape was ecologically diverse and a highly productive biome (Miracle 1995, 1996, 2007b; Oros Sršen et al. 2026). Situated on the northwestern edge of the plain, Istria was not an isolated peninsula as it is today, but an integral part of a continuous terrestrial corridor linking Italian and Balkan refugial zones. This strategic position would have facilitated the movement of both animals and human hunter‐gatherer groups, contributing to a dynamic ecological and cultural landscape during the coldest phases of the Pleistocene (Borić and Cristiani 2016; Forenbaher 2020; Miracle 2007b; Peresani et al. 2021; Ruiz‐Redondo et al. 2022; Whallon 2007).

Documenting genetic diversity in populations that survived glacial periods is essential for reconstructing the evolutionary pathways that shaped Holocene and modern cattle. Glacial refugia acted as reservoirs of genetic variation from which postglacial populations expanded, meaning that the lineages preserved during the Last Glacial Maximum represent the ancestral genetic foundation of later cattle populations. Without precise insight into the genetic structure of these refugial populations, it is difficult to determine the gene pools from which postglacial lineages originated and to identify the oldest haplogroups that played a role in later diversification and local adaptations (Cubric‐Curik et al. 2022). In Europe, only seven ice‐age aurochs sequences are currently available (Rossi et al. 2024), of which six are directly dated. These include two sequences from an extinct lineage (haplogroup G) from the Rhine region (older than 45 ka), one specimen from the Caucasus assigned to haplogroup K (14 ka), and three specimens from Italy, all belonging to haplogroup P (18, 14, 13 ka). Every new ice‐age specimen is thus critical for refining our understanding of population structure, refugial isolation, and long‐term evolutionary patterns, especially in southeastern Europe, which remains poorly studied. Most research has focused on the Iberian refugium (Rossi et al. 2024), leaving Italy and the Balkans largely unexplored. To fill this gap we sequenced two Pleistocene aurochs from the archaeological site of Šandalja in Istria (situated at the intersection of both regions) and sought to determine whether they were part of an undifferentiated southern European metapopulation or represented a regionally distinct population. The resulting genomes represent the first directly dated aurochs ( *Bos primigenius* ) from the eastern Adriatic region, providing unique insights into the population structure of the Apenine‐Adriatic refugium. These data are of key importance for reconstructing population movements, genetic diversity, and phylogeographic patterns of aurochs during the LGM and postglacial periods. By contributing new evidence from a previously unsampled geographic area, this study enhances our understanding of bovine evolutionary history and clarifies the role of the Great Adriatic Plain as an important refugial and migratory landscape in the Late Pleistocene. More broadly, our findings will help refine broader models of megafaunal biogeography and deepen our knowledge of how glacial landscapes shaped the ancestry of modern species.

## 2 Material and Methods

### 2.1 Archaeological Context and Material

The site ‘Šandalja’ is the largest of at least four fossil‐bearing localities discovered in a limestone quarry four km northeast of the city of Pula, Croatia (44° 52′ 57″ N, 13°53′48″ E, elevation 72 m/sl Figure 1). The material discussed in this paper comes from a fossil cave, Šandalja II (hereafter ‘Šandalja’) that was exposed by limestone quarrying in 1962; at the time of discovery over 8 m of Upper Pleistocene sediments were exposed in an oval‐shaped profile (Malez 1963). Šandalja was subsequently excavated from 1962 to 1989 (Miracle 1995). Upper Palaeolithic stone tools from ‘Aurignacian’ and Epigravettian technocomplexes, alongside associated ice‐age animal remains, were found in a sequence of seven major sedimentary units (Layers B–H) spanning from c. 30,000–12,000 cal BP (Malez and Vogel 1969). Recent AMS 14C dates have extended the sequence to c. 45,000 cal BP (Ruiz‐Redondo et al. 2024), although a series of directly dated animal bones have called into question the integrity of the stratigraphic sequence (Miracle and Brajković 2013; Oros Sršen et al. 2014; Richards et al. 2015; Ruiz‐Redondo et al. 2024).

Here we present results for two specimens identified as aurochs from Šandalja, SA5 (HRV_SA5_11612) and SA6 (HRV_SA6_14757). SA5 is from a right carpal 2 + 3 bone (Inventory: Sa‐2000, from layer ‘E’). HRV_SA6_14757 is from a right carpal 4 bone (Inventory: Sa‐2151, from layer ‘B/g’). These bones were chosen because they were available, complete, and visually appeared to be promising for the preservation of biomolecules. The layer labels suggest that HRV_SA5_11612 is older than HRV_SA6_14757, by perhaps as much as 10,000 years. Direct AMS ^14^C dating of these samples, however, provides further evidence of stratigraphic mixing at the time of excavation or during the later curation of the remains; HRV_SA5_11612 is dated to 11,825–11,361 cal BP (FTMC‐VK76‐1), while HRV_SA6_14757 is dated to 14,847–14,163 cal BP (FTMC‐VK76‐2). Further information about these dates is presented in Table S1 and Figure S1.

### 2.2 Laboratory Procedures

All ancient bone drilling was performed in dedicated clean‐room facilities at the LAZAG Laboratory, University of Zagreb Faculty of Agriculture, where approximately 50–100 mg of bone powder was obtained from each specimen. DNA extraction and library reparation wereconducted in dedicated ancient DNA clean laboratories at GeoGenetics, University of Copenhagen using semi‐automated workflows. Following ancient DNA protocols (McColl et al. 2024), double‐stranded dual‐indexed Illumina libraries (Meyer and Kircher 2010) were constructed from each extract and sequenced paired‐end (2 × 100 bp) on an Illumina NovaSeq 6000 platform at the GeoGenetics Sequencing Core (University of Copenhagen, DK; Table S1).

### 2.3 Sequencing Read Processing

Raw paired‐end reads from the two Šandalja aurochs (HRV_SA5_11612 and HRV_SA6_14757) were quality‐checked with FastQC v0.12.1 and processed with AdapterRemoval v2.3.4 (Schubert et al. 2016) using ‐‐collapse ‐‐trimns ‐‐trimqualities ‐‐minquality 25 ‐‐minlength 18, producing collapsed reads as input for both nuclear and mitochondrial mapping.

### 2.4 Alignment and Post‐Alignment Processing

Collapsed reads were mapped to the *Bos taurus* reference genome ARS‐UCD1.2 (with the Y chromosome added; ARS‐UCD1.2_Btau5.0.1Y) using BWA v0.7.18 (Li and Durbin 2009) with the aln algorithm and ancient‐DNA parameters (‐l 1024 ‐n 0.01 ‐o 2), followed by bwa samse. Read groups were added with Picard AddOrReplaceReadGroups (v3.3.0) (Broad Institute 2019) and PCR duplicates removed with Picard MarkDuplicates (REMOVE_DUPLICATES = true). Local indel realignment was performed with GATK v3.8 (RealignerTargetCreator + IndelRealigner) (McKenna et al. 2010). Reads with mapping quality below 25 were filtered out (samtools view ‐q 25; Danecek et al. 2021). Post‐mortem damage was verified with mapDamage2 v2.2.3 (Jónsson et al. 2013) (Figure S2) and per‐sample coverage assessed with Qualimap v2.2.1. (Okonechnikov et al. 2016). Endogenous DNA content (primary mapped reads/total collapsed reads) was 26.1% for HRV_SA5_11612 and 28.3% for HRV_SA6_14757; after MAPQ ≥ 25 filtering, 7.2% and 6.2% of reads were retained, respectively. Final autosomal coverage was 0.078× (HRV_SA5_11612) and 0.052× (HRV_SA6_14757).

### 2.5 Molecular Sex Determination

Molecular sex of the two Šandalja aurochs (HRV_SA5_11612 and HRV_SA6_14757) was determined from the mapped reads using three ratios: Ry, the fraction of sex‐chromosome reads mapping to the Y (Skoglund et al. 2013); Rx1, X‐chromosome reads relative to chromosome 1 (Ginja et al. 2023); and Rx2, X‐chromosome reads relative to chromosome 2. As chromosome 2 (136.23 Mbp) closely matches the X chromosome (139.01 Mbp) in length, Rx2 is expected to be at least as reliable as Rx1. A final assignment (XX, XY, or ‘not assigned’) was made jointly from the three ratios (Table S1). For the comparative samples, sex was taken as reported in the respective source publications (Rossi et al. 2024; Erven et al. 2024 for Bed3; Park et al. 2015 for CPC98).

### 2.6 Mitochondrial DNA Consensus Generation

In parallel, the same collapsed reads were mapped to the *Bos taurus* mitochondrial reference V00654 (Anderson et al. 1982) using BWA v0.7.18 with the same parameters as the nuclear alignment, followed by read‐group assignment, duplicate removal (Picard MarkDuplicates) and MAPQ ≥ 25 filtering. Mean mitochondrial coverage (V00654 mapping) was 8.11× for HRV_SA5_11612 and 3.48× for HRV_SA6_14757, with 99.98% and 93.09% of the 16,338‐bp mitogenome covered at ≥ 1×, respectively. Per‐sample mitochondrial consensus sequences were generated with ANGSD v0.940 (Korneliussen et al. 2014) using ‐doFasta 2 ‐doCounts 1 ‐setMinDepth 3 ‐minQ 20 ‐minMapQ 30.

### 2.7 Mitogenome Phylogenetic Analysis

Bayesian phylogenetic inference of whole mitochondrial consensus sequences was performed in BEAST X v10.5.0 (Baele et al. 2025), using the BEAGLE library v4.0.1 (Ayres et al. 2019; Gangavarapu et al. 2024). Consensus sequences from 167 taxa were aligned against the *Bos taurus* mitochondrial reference (GenBank V00654.1; Anderson et al. 1982) using MAFFT (Katoh and Standley 2013), yielding a 16,349‐bp multiple‐sequence alignment that was imported into BEAUTi. For ancient samples, tip dates were specified as uniform priors bounded by the 95% confidence interval of the calibrated radiocarbon date; modern samples were assigned a tip date of zero.

The complete mitogenome was modelled as a single, unpartitioned alignment under the General Time Reversible substitution model (Lanave et al. 1984) with four discrete gamma‐distributed rate categories (Yang 1994) and a proportion of invariant sites (GTR + T_4_ + I). Among‐lineage rate variation was modelled with an uncorrelated lognormal relaxed molecular clock (Drummond et al. 2006), and a coalescent constant‐size population (Kingman 1982; Drummond et al. 2002) was used as the tree prior. Two independent MCMC chains of 800,000,000 generations each were run, sampling every 80,000 states. Convergence was assessed in Tracer v1.7 (Rambaut et al. 2018); runs were combined with LogCombiner after all parameters reached ESS > 200, discarding the first 10% as burn‐in. A maximum clade credibility (MCC) tree with median node heights was summarised in TreeAnnotator; 95% HPD bars are shown only on nodes with posterior probability ≥ 0.9. The MCC tree was visualised in FigTree v1.4.4 (Rambaut 2018). Haplogroup assignment for each sample was determined from its position on this tree. Tip labels in the MCC tree follow the convention ISO‐3166 country code_specimenname_mean calibrated age (years BP), and the same labels are used throughout the manuscript; the full mapping is provided in Table S1.

### 2.8 Median‐Joining Network Analysis

For the mitogenome haplotype‐diversity analysis, a subset of 34 samples assigned to haplogroup P by Bayesian phylogenetic inference was selected. Only complete mitochondrial consensus sequences (16,349 bp) were considered, and one sample with an excessive proportion of ambiguous bases (N) was excluded (UZB_Kok1_3500), leaving 33 sequences. Missing positions in HRV_SA6_14757 were imputed by reference to the aligned positions of the closely related sample HRV_SA5_11612 (7522 bp imputed in total); positions missing in both consensus sequences were retained as missing data. To reduce noise, sites at which a variant occurred exclusively in HRV_SA5_11612 or HRV_SA6_14757 and was absent in all other samples were treated as singletons and removed. After excluding sites containing indel gaps, missing data or singletons, the final alignment comprised 16,291 bp. A median‐joining network (MJN; Bandelt et al. 1999) of the whole‐mitogenome consensus sequences (16,291 bp) was then inferred in PopART (Leigh and Bryant 2015) to visualise the haplogroup P haplotypes.

### 2.9 Nuclear DNA Analysis

Given the low coverage of both samples, pseudohaploid genotypes were called with ANGSD v0.940 (Korneliussen et al. 2014) using ‐doHaploCall 1 ‐doCounts 1 ‐GL 1 ‐minMapQ 30 ‐minQ 25 ‐trim 6 ‐uniqueOnly 1 ‐remove_bads 1 ‐C 50, restricted to the autosomal transversion SNP positions of Rossi et al. (2024). Reference alleles were taken from our reference assembly; reference/alternate allele swaps in the Rossi VCF (~2.9% of positions) were harmonised with bcftools norm ‐‐check‐ref ws (Danecek et al. 2021).

HRV_SA5_11612 and HRV_SA6_14757 genotypes were merged using PLINK v1.9 (Purcell et al. 2007) with 20 ancient aurochs samples provided by Rossi et al. (2024) (full sample IDs and metadata Table S1), yielding 22 ancient aurochs and 5,516,927 LD‐pruned (‐‐indep‐pairwise 50 5 0.5) transversion‐only SNPs. Following Cai et al. (2026), SNPs falling in 35‐bp windows of low mappability (Heng Li's SNPable filter, c < 3, *r* = 0.5), 10‐kbp windows of extreme GC content (top or bottom 2.5%), 10‐kbp windows with high repeat content (top 5%), or 10‐kbp windows containing ≥ 10 uncalled (N) bases were excluded. The final dataset comprised 1,634,099 SNPs across 22 aurochs.

Pairwise identity‐by‐state distances (1–IBS) were computed in PLINK v1.9 (‐‐distance 1‐ibs square), using only positions where both samples carried a called genotype. Coverage‐driven bias was assessed with a Mantel test (Mantel 1967) between the IBS matrix and the matrix of estimated shared genotyped positions per pair (R package vegan; Oksanen et al. 2024). There was no significant positive association between IBS distance and reduced position sharing (Spearman *r* = −0.43, *p* = 0.99; 9999 permutations), that is, pairs sharing fewer genotyped positions did not show systematically inflated distances. The negative coefficient is attributable to the single HRV_SA5_11612–HRV_SA6_14757 pair, which shares only ~2,800 genotyped positions and shows an anomalously low distance; this pair is therefore treated as uninformative throughout. IBS matrices were visualised as heatmaps with hierarchical clustering (complete linkage; pheatmap; Kolde 2025) and as midpoint‐rooted Neighbour‐Joining trees (ape; Paradis and Schliep 2019). Multidimensional scaling (MDS) on the IBS matrix was performed with PLINK v1.9 (‐‐cluster ‐‐mds‐plot 10), following Rossi et al. (2024). The Spearman correlation between sample‐wise MDS Euclidean distances (across the first 10 coordinates) and per‐sample genotype missingness was negligible for both Šandalja aurochs (HRV_SA5_11612 *ρ* = −0.02; HRV_SA6_14757 *ρ* = −0.20), confirming that their MDS positions are not driven by differential coverage.

## 3 Results

### 3.1 Archaeological and Genomic Characterisation of the Samples

We successfully sequenced DNA extracted from two carpal bones, HRV_SA5_11612 and HRV_SA6_14757, excavated from the prehistoric site of Šandalja near Pula (Istria), Figure 1.

Both specimens were identified as Pleistocene aurochs ( *Bos primigenius* ) based on their morphology and the stratigraphic context of the site (Miracle 1995). Direct radiocarbon dating performed at the Vilnius Radiocarbon Laboratory corroborates this assessment, indicating that HRV_SA6_14757 represents the older specimen, with a calibrated age range of 14,847–14,163 cal BP, whereas HRV_SA5_11612 is approximately 3000 years younger, dating to 11,825–11,361 cal BP (see Table S1; Figure S1). Genomic sex determination, based on the ratio of reads mapped to the X chromosome relative to chromosome Y, 1 and 2, identified both specimens as female (Table S1). The samples yielded DNA of sufficient quality for both nuclear and mitochondrial genome analyses, showing characteristic ancient DNA signatures, including fragmentation, terminal cytosine deamination, and average read lengths. Endogenous DNA content after quality filtering was 6.2% for HRV_SA6_14757 and 7.2% for HRV_SA5_11612, resulting in genome‐wide average coverages of 0.052× and 0.078×, respectively. This is in the range of genome coverages sequenced in other Pleistocene aurochs found in Europe (Rossi et al. 2024). Mitochondrial genomes were also successfully retrieved, with 93.1% coverage at 3.5× depth for HRV_SA6_14757 and 99.9% coverage at 8.1× depth for HRV_SA5_11612.

### 3.2 Mitogenome Analyses

**FIGURE 2:**
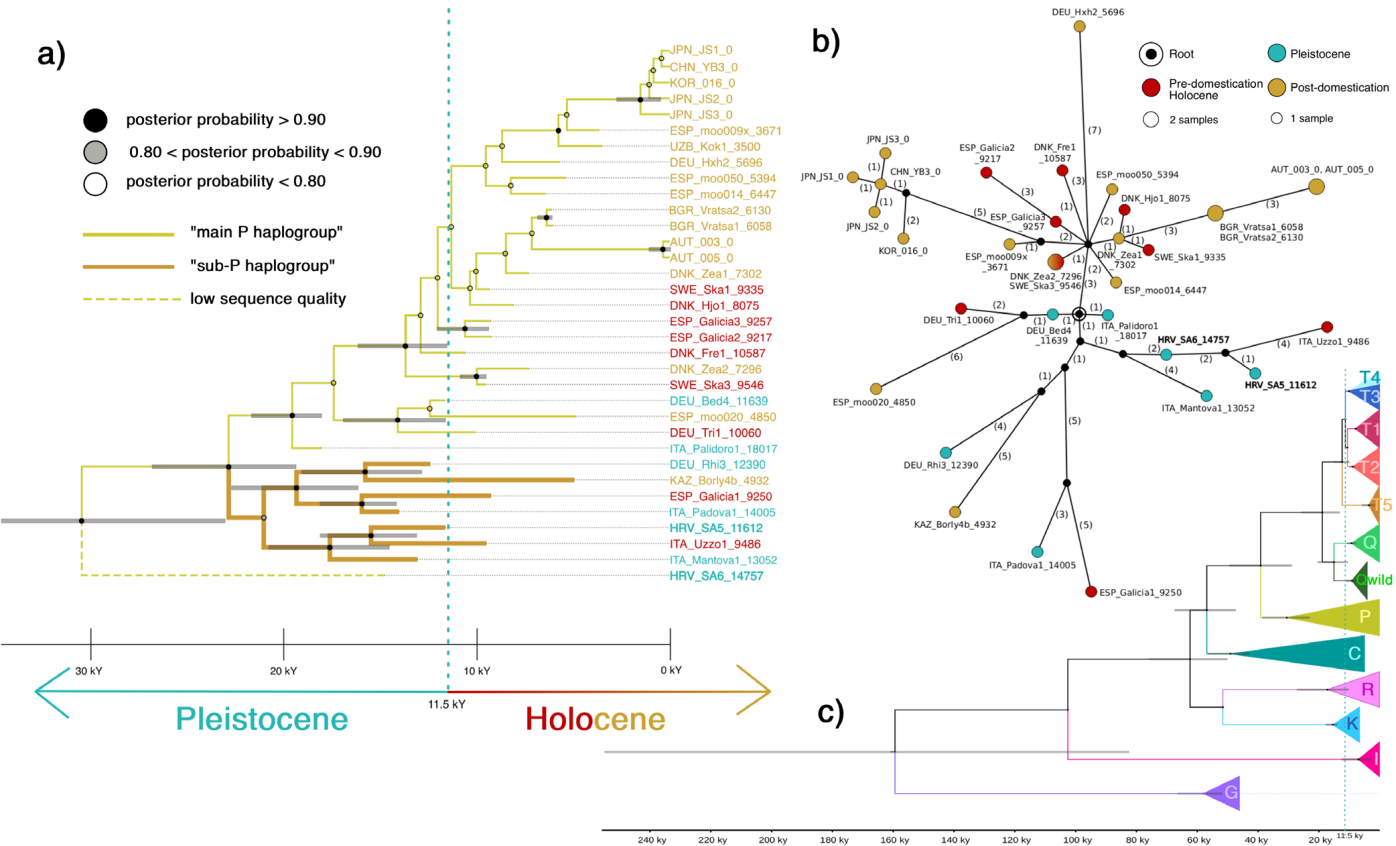
Complete mitogenome phylogenetic reconstruction and haplotype‐network analysis of European aurochs and modern cattle. (a) Partial Bayesian phylogenetic tree showing P haplotypes; (b) Median‐joining network illustrating relationships among P haplotypes; and (c) Bayesian phylogenetic tree.

Results of the Bayesian phylogenetic analysis (167 sequences) and the Median‐Joining network (33 mitogenomes) are presented in Figure 2a–c. The BEAST tree places the two Istrian aurochs (HRV_SA6_14757 and HRV_SA5_11612) unambiguously within haplogroup P (Figure 2a), clustering them with 32 previously published *Bos primigenius* sequences (Bro‐Jørgensen et al. 2018; Günther et al. 2025; Park et al. 2015; Rossi et al. 2024; Verdugo et al. 2019) and with several ancient and modern *B. taurus* individuals (Cubric‐Curik et al. 2022; Mannen et al. 2020). This placement reinforces the well‐documented continuity of haplogroup P from Late Pleistocene aurochs into present‐day cattle. Within haplogroup P, HRV_SA5_11612 is placed in a phylogenetically distinct lineage, here provisionally referred to as the ‘alternative’ P sub‐haplogroup. This lineage is clearly separated from the ‘main’ P sub‐haplogroup, which includes historical and modern taurine cattle samples (Figure 2a). The node separating the ‘alternative’ and ‘main’ P lineages is strongly supported, with a posterior probability of 1. Molecular dating places this split at approximately 26.8–19.4 ky BP, based on the 95% highest posterior density interval, predating the diversification of the ‘main’ P lineage reported by Mannen et al. (2020) and Cubric‐Curik et al. (2022).

The BEAST tree shows a further division within our ‘alternative’ sub‐haplogroup P into two suggestively separated clusters (0.80 < posterior probability < 0.90). The first consists of the HRV_SA5_11612 aurochs together with two Italian Pleistocene/early Holocene individuals (Mantova1, 13,052 BP; Uzzo1, 9486 BP). The second cluster contains Italian (Padova1, 14,005 BP), Spanish (Galicia1, 9250 BP), and German aurochs (Rhi3, 12,390 BP), alongside one mid‐Holocene Kazakh individual (Borly4b, 4932 BP). The close genetic clustering of the Istrian sample with most Italian samples is consistent with shared ancestry among aurochs associated with the Great Adriatic Plain (GAP) and may reflect a genetically coherent regional population during the Late Pleistocene and early Holocene. The second Istrian aurochs, HRV_SA6_14757, was generally placed outside both the ‘main’ and ‘alternative’ P sub‐haplogroups. However, its position varied across analytical models, with some reconstructions placing it within, or close to, the ‘alternative’ P sub‐haplogroup.

The Median‐Joining (MJ) network constructed from an informativeness‐based subset of P haplotypes and using the corrected HRV_SA6_14757 sequence (imputed at missing sites using the closest sequence, HRV_SA5_11612) independently corroborates this interpretation (Figure 2b). The MJ network reconstructs a clear progression from Late Pleistocene haplotypes through pre‐domestication Holocene lineages and onward to post‐domestication and modern cattle. Except for KAZ_Borly4b_4932, which retains a deep position within the ‘alternative’ P sub‐haplogroup radiation, the network sharply separates early Pleistocene branches from Holocene domestic‐associated haplotypes. We were surprised that the modern P haplotype found in Murbodner cattle (Cubric‐Curik et al. 2022) connects to the root of haplogroup P via a series of Holocene aurochs from Bulgaria (Vratsa1, 6058 BP; Vratsa2, 6130 BP) and Denmark (Zea1, 7302 BP). This sequential accumulation of mutations suggests a structured postglacial expansion of P maternal lineages across Europe, tracing their movement from western Europe through northern routes and onward into eastern Europe.

### 3.3 Autosomal Genomic Analyses

Our autosomal genomic analyses were based on pairwise identity‐by‐state (IBS) distances calculated among the samples analysed in this study and a reference dataset of aurochs from Rossi et al. (2024). This approach was adopted because the quality and completeness of autosomal genomic data in our samples were generally low. Across all pairwise comparisons, the number of shared non‐missing SNPs varied widely, reflecting substantial heterogeneity in data coverage among individuals. Specifically, shared SNP counts ranged from 1089 to 1,129,418 across sample pairs, with a median of 253,630 and a 5%–95% quantile range of 9,147–1,017,466. Pairs involving the Croatian samples exhibited lower, but relatively consistent, levels of SNP overlap. For HRV_SA5_11612, the number of shared non‐missing SNPs with other individuals ranged from 1671 to 36,997 (median = 29,752), whereas for HRV_SA6_14757 the corresponding range was 1089 to 16,702 (median = 13,735). The HRV_SA5_11612 and HRV_SA6_14757 pair shared 2745 non‐missing SNPs. Despite this pronounced heterogeneity in SNP overlap, IBS‐based similarity patterns were stable across analyses, particularly with respect to our main hypothesis concerning the existence of a genetically coherent GAP aurochs population.

**FIGURE 3:**
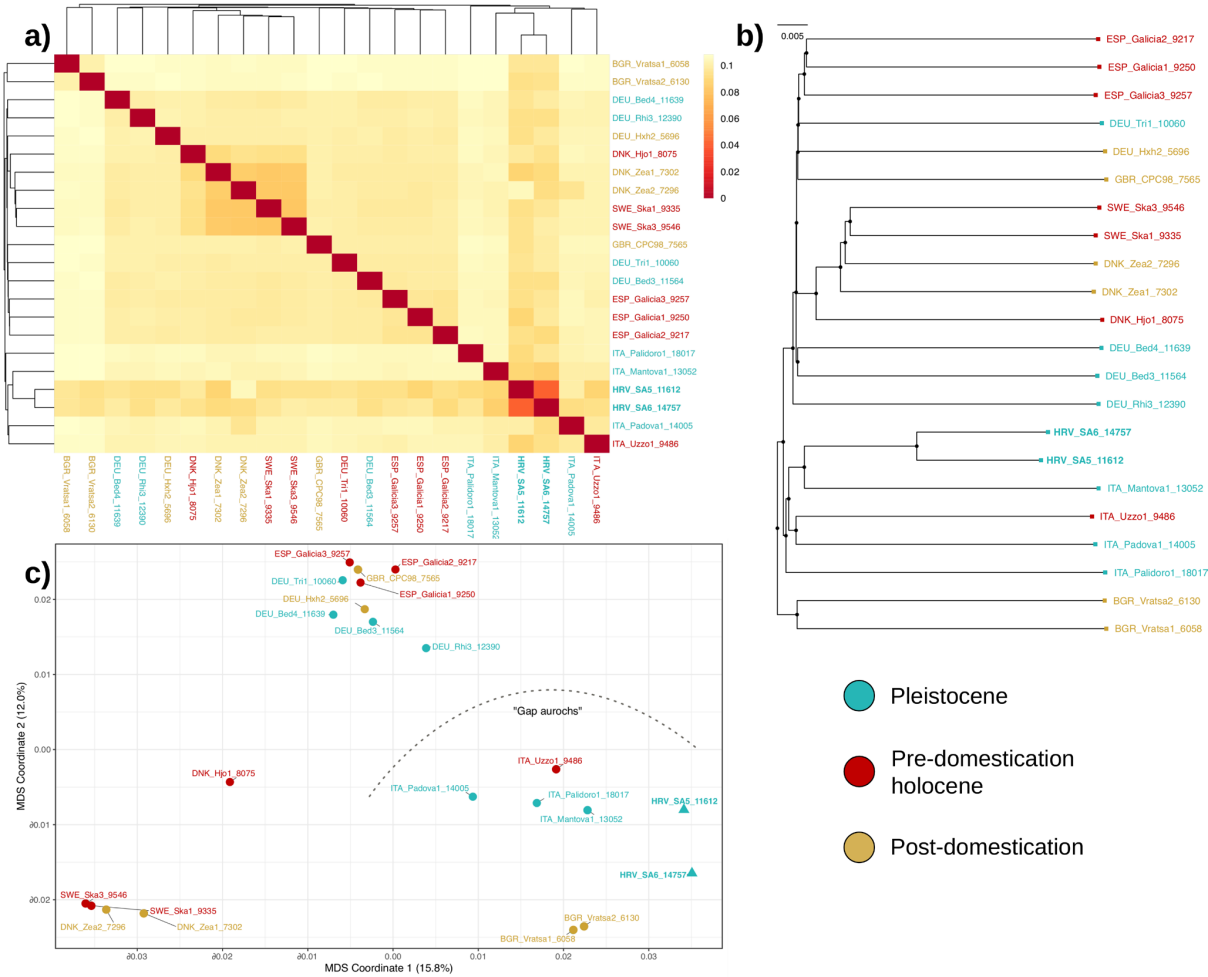
Genome‐wide IBS‐derived genetic distances among ancient aurochs. Pairwise genetic distances, calculated as 1−IBS, were estimated among 22 ancient aurochs, including HRV_SA5_11612, HRV_SA6_14757, and 20 samples from Rossi et al. (2024), using 1,634,099 LD‐pruned, damage‐masked, transversion‐only SNPs. Distances are visualised as (a) a heatmap, (b) a midpoint‐rooted Neighbour‐Joining tree, and (c) multidimensional scaling (MDS) of the distance matrix.

Our IBS‐distance analyses are presented in Figure 3. Overall, the mitogenome‐ and autosome‐based analyses revealed broadly concordant patterns, but also several notable differences (Figures 2 and 3). In contrast to the mitochondrial results, the autosomal analyses showed a stronger correspondence with geographic origin. For example, the Spanish aurochs samples (Galicia1–Galicia3) formed a distinct autosomal cluster in both the heatmap and the Neighbour‐Joining tree (Figure 3a,b), whereas the mitogenome‐based analysis placed Galicia1 closer to the GAP‐associated aurochs lineage. Similarly, the Danish aurochs samples clustered together in the autosomal analyses, despite being assigned to different groups within the ‘main’ P mitochondrial haplogroup. Importantly, the autosomal results provide additional support for the presence of a genetically coherent aurochs population associated with the Great Adriatic Plain (GAP). In the IBS‐distance heatmap, all Italian and Istrian aurochs, with the exception of ITA_Palidoro1_18017, formed a relatively homogeneous group (Figure 3a). In the midpoint‐rooted Neighbour‐Joining tree and MDS plot, HRV_SA6_14757 and ITA_Palidoro1_18017 were also positioned within or close to the GAP‐associated cluster (Figure 3b,c).

## 4 Discussion

During cold phases of the Pleistocene, aurochs populations retreated into several major glacial refugia, including the Istrian region, which in the Late Glacial formed a low rise on the edge of the Great Adriatic Plain. Freshwater was likely available locally from springs and other karstic features (Oros Sršen et al. 2026), while significant wetlands have been reconstructed approximately 30 km to the northeast in the Kvarnerić depression and 50–100 km to the south, where the Palaeo‐Po River and its tributaries flowed (Figure 1). There is considerable evidence from Šandalja's faunal assemblages and from stable isotopic analyses of human remains for the exploitation of freshwater habitats during the Late Pleistocene (Miracle 1995; Paunović 1984; Richards et al. 2015). Šandalja has a large and diverse assemblage of mammal remains, with 11,990 identified specimens (NISP) of hare‐sized and larger mammals from 30 species (Miracle 1995, 2007b; Richards et al. 2015). Aurochs is the best‐represented taxon (NISP = 2835), and these remains present abundant evidence of human processing and consumption (Miracle 1995, 1996, 2007b). The faunal evidence from Šandalja indicates that southern Istria, and by extension the Great Adriatic Plain, provided excellent habitat for aurochs during the Late Glacial period (Miracle 2007b).

Both Šandalja specimens are securely identified as Pleistocene *Bos primigenius* based on morphology, stratigraphy, and direct radiocarbon dating (Miracle 1995), and they exhibit endogenous DNA content and genome coverage comparable to other sequenced Pleistocene aurochs (Rossi et al. 2024). Bayesian phylogenetic analyses place HRV_SA5_11612 and HRV_SA6_14757 unambiguously within mitochondrial haplogroup P, the dominant European aurochs lineage that persisted at low frequencies into modern taurine cattle (Cubric‐Curik et al. 2022; Mannen et al. 2020; Park et al. 2015; Rossi et al. 2024; Verdugo et al. 2019). One Šandalja sample falls within an early‐diverging, phylogenetically distinct ‘alternative’ P sub‐haplogroup, in contrast to the majority of previously reported P lineages that cluster within the ‘main’ P sub‐haplogroup associated with Holocene aurochs and domestic *Bos taurus* . The BEAST phylogeny further resolves this ‘alternative’ P sub‐haplogroup into two well‐supported clusters. One cluster comprises the Šandalja sample together with two Italian Pleistocene/early Holocene individuals (ITA_Mantova1_13052 and ITA_Uzzo1_9486), while the second cluster (0.80 < posterior probability < 0.90) includes additional Italian, Spanish, and German aurochs, as well as mid‐Holocene individuals from Kazakhstan (Bro‐Jørgensen et al. 2018; Günther et al. 2025; Park et al. 2015; Rossi et al. 2024; Verdugo et al. 2019). The Median‐joining network constructed from an informativeness‐based subset of P haplotypes independently corroborates this interpretation. Using the corrected HRV_SA6_14757 sequence (imputed with HRV_SA5_11612), the network reconstructs a temporal progression from Late Pleistocene haplotypes, through pre‐domestication Holocene aurochs, to post‐domestication and modern cattle. With the notable exception of KAZ_Borly4b_4932, which retains a deep position within the ‘alternative’ P radiation, early Pleistocene branches are sharply separated from Holocene domestic‐associated haplotypes. This pattern supports a scenario in which only a restricted subset of P lineages survived the demographic and selective filters associated with postglacial environmental change and cattle domestication (Cubric‐Curik et al. 2022; Mannen et al. 2020; Rossi et al. 2024). The position of the modern P haplotype found in Murbodner cattle is particularly interesting in this context (Cubric‐Curik et al. 2022). It connects to the root through a chain of Holocene aurochs from Bulgaria, Denmark, and Spain, rather than through the deeply diverging Adriatic lineage. This structured accumulation of mutations suggests a postglacial expansion of P lineages along northern, and then eastern routes, and implies that the GAP population documented at Šandalja contributed little, if at all, to the maternal ancestry of modern European cattle. Whether this reflects true extinction of the Adriatic maternal line, demographic swamping by expanding domestic populations, or stochastic lineage sorting in small Holocene refugial populations remains unresolved.

At the nuclear genomic level, the analysed aurochs displayed a pronounced geographic pattern, suggesting that autosomal variation largely reflects regional population structure. This pattern was visible across the IBS‐distance heatmap (Figure 3a), the midpoint‐rooted Neighbour‐Joining tree (Figure 3b), and the MDS plot (Figure 3c). Samples from the same or neighbouring regions tended to group together, with Spanish, Scandinavian, German, Bulgarian, and Italian aurochs forming geographically coherent clusters. Most importantly, the Istrian samples from Šandalja showed a clear genetic affinity with Italian aurochs, especially those associated with the northern Adriatic and Po Valley region. This clustering supports the interpretation that the Istrian and Italian specimens may have belonged to a genetically connected aurochs population associated with the Great Adriatic Plain. Although the low coverage of some genomes requires cautious interpretation, the consistency of this pattern across different IBS‐based visualisations strengthens the evidence for regional structure among Late Pleistocene and Early Holocene aurochs populations. The emergence of an ‘alternative’ mitochondrial sub‐haplogroup between approximately 26.8–19.4 ky BP predates the radiation of the ‘main’ P clade that was ultimately introgressed into modern domestic cattle (Cubric‐Curik et al. 2022; Mannen et al. 2020). Unfortunately, owing to the low informativeness and limited coverage of the autosomal genomic data, we were unable to quantify the contribution of Istrian aurochs to modern cattle genomes. This limitation underscores the need for additional high‐coverage sequencing of samples from the GAP population to enable more conclusive inference of autosomal ancestry relationships and their potential impact on the genetic variability of modern cattle. Given the scarcity of directly dated ice‐age aurochs genomes (Rossi et al. 2024), the Šandalja sequences fill an important gap in the temporal and phylogenetic coverage of haplogroup P, firmly establishing the Adriatic Basin as an important region of Pleistocene aurochs diversity.

Overall, the observed patterns align well with palaeogeographical reconstructions showing that sea‐level lowering during the Last Glacial Maximum exposed a vast lowland—the Great Adriatic Plain—connecting Istria and the Dinaric hinterland with the Po Valley (Maselli et al. 2014). Archaeological evidence further highlights the GAP as a key biogeographic corridor linking Italian and Balkan refugial zones, facilitating the movement of both large mammals and human hunter‐gatherer groups (Forenbaher 2020; Miracle 1995; Peresani et al. 2021). The phylogenetic cohesion of the Adriatic lineage identified here is therefore consistent with an ice‐age GAP refugial population that persisted within this continuous landscape until the early Holocene.

Placed in this broader palaeoenvironmental context, the Šandalja genomes add to growing evidence that European aurochs populations were already genetically structured well before domestication, with distinct mitochondrial lineages—such as haplogroups G, C, K, R, E and P—occupying different regions and refugia across Eurasia (Bonfiglio et al. 2010; Brunson et al. 2023; Chen et al. 2024; Ginja et al. 2023; Hou et al. 2024; Rossi et al. 2024; Verdugo et al. 2019). These results identify the Apenine‐Adriatic region as a previously underappreciated refugial zone, complementing the better‐studied Iberian refugium and underscoring the importance of southeastern Europe in the late‐glacial biogeography of large herbivores.

Furthermore, our results from Šandalja reveal an informative pattern in Pleistocene Italian aurochs samples. Specimens from the Po Valley (ITA_Mantova1_13052 and ITA_Padova1_14005), a northwestern extension of the Great Adriatic Plain, cluster closely with the Istrian sequences and are broadly contemporaneous, dating to approximately 15–12 ka BP. In contrast, ITA_Palidoro_18017, located on the western side of the Apennines (Figure 1), is older, dating to around 18 ka BP. Notably, its maternal lineage clusters with western European aurochs, whereas its nuclear genome shows affinity to the other Italian samples (Rossi et al. 2024).

There is substantial archaeological evidence for cultural connections between southern France and northwestern Italy during the Upper Palaeolithic, particularly prior to the Last Glacial Maximum (LGM) (Gamble 1986; Mussi 2000, 2001). The Alpine chain approaches the Tyrrhenian coast between Nice and Genoa, and during Pleistocene stadial periods the coastal plain was narrow, as it is today. This geography likely did not constitute a significant barrier to the movement of either Pleistocene aurochs or Palaeolithic human populations. One interpretation is that ITA_Palidoro_18017 represents a residual eastern outlier of a pre‐LGM aurochs gene pool that extended across the Franco‐Cantabrian region. Alternatively, and arguably more biologically intuitive given the autosomal clustering observed in the Neighbour‐Joining tree and MDS analysis, ITA_Palidoro_18017 may represent an ancestral lineage of a refugial aurochs population primarily associated with the Adriatic Basin. Testing these hypotheses requires directly dated Pleistocene aurochs genomes from France and the Iberian Peninsula.

Another unexpected result from the Italian material is the clustering, supported by both mitogenome and autosomal genomic analyses, of an early Holocene aurochs from Sicily (ITA_Uzzo1_9486) with the late glacial sequences from Šandalja. Uzzo1 is 2000 years younger than HRV_SA5_11612; it suggests that the GAP gene pool during the late glacial period was widely distributed, from the northern Adriatic, down the length of Italy, across the straits of Messina, to the northwestern corner of Sicily. There are further interesting parallels with humans and their cultures. Several thousand years later (c. 7500 yBP) there are genetic and cultural similarities between neolithic people from Uzzo Cave (source of ITA_Uzzo1_9486) and Dalmatia, on the eastern Adriatic coast (Yu et al. 2022), although in this case the similarity arises from shared ancestors who brought farming and herding to both regions from places further to the east (northwestern Greece, Anatolia, and the ‘fertile crescent’).

We acknowledge that some degree of bias arising from the low quality and limited completeness of the genomic data cannot be entirely excluded. Nevertheless, when considered collectively, all analyses consistently indicate that the Istrian aurochs form part of a genetically coherent aurochs population associated with the Great Adriatic Plain. The Šandalja specimens represent some of the earliest sequenced European aurochs genomes, most notably HRV_SA6_14757 (the second oldest known representative of the P mitochondrial haplogroup in Europe) and therefore provide valuable insight into pre‐domestication genomic diversity. These lineages preserve a distinct Pleistocene mitochondrial signature and nuclear genomic affinity that persisted into the early Holocene in Sicily. Whether this lineage contributed to the genetic makeup of modern cattle remains unresolved and will require further targeted investigation.

## 5 Conclusion

By integrating archaeological context, direct radiocarbon dating, and ancient whole‐genome sequencing, this study provides new insight into the genetic structure of Late Pleistocene aurochs from the Great Adriatic Plain. Mitochondrial and autosomal genomic evidence support the presence of a genetically coherent, early‐diverging Adriatic–Italian aurochs population that persisted in this refugial landscape during the Late Pleistocene and early Holocene. The Šandalja specimens, including HRV_SA6_14757, one of the oldest known European representatives of mitochondrial haplogroup P, highlight the extent to which Pleistocene aurochs diversity in southeastern Europe remains underrepresented. Although the available data suggest that this Adriatic lineage may have contributed little, if at all, to the ancestry of modern cattle, this remains a hypothesis that requires testing through additional high‐coverage genomes and broader comparative datasets. More broadly, our results support the interpretation that the now‐submerged Great Adriatic Plain acted as an important refugial landscape and biogeographic corridor linking the Apennine and Balkan Peninsulas, with wider implications for the evolutionary history of European large mammals.

## Author Contributions

Conceptualisation: V.C.‐C., P.T.M., I.C. Data curation: V.C.‐C., P.T.M., I.C., M.F., D.N. Analysis: D.N., M.F., Z.M., I.K., V.C.‐C., P.T.M., A.C., M.B. Funding acquisition: V.C.‐C., I.C., P.T.M. Investigation: V.C.‐C., P.T.M., I.C. Methodology: V.C.‐C., P.T.M., I.C., A.P., E.W., S.R., M.F., J.S. Visualisation: P.T.M., I.C., I.K., M.F., Z.M., D.N. Writing – original draft: V.C.‐C., P.T.M., I.C. Writing – review and editing: all authors.

## Funding

This work was supported by grants from Croatian Science Foundation under project number HRZZ IP‐2022‐10‐8926 (Gabridge) and project KK.01.1.1.06.0002 (LazAg).

## Conflicts of Interest

The authors declare no conflicts of interest.

## Supporting information


**Table S1:** Information on Šandalja archaeological samples and published samples used in the analyses. Sheet 1, information on Šandalja archaeological samples; Sheet 2, radiocarbon dating results of Šandalja samples; Sheet 3, samples used for BEAST analysis; Sheet 4, samples used for nuclear DNA analysis; Sheet 5, samples used for Median‐joining network analysis; Sheet 6, sex determination results of Šandalja samples.

**Figure S1:** Radiocarbon date calibration curves of the two Šandalja samples.

**Figure S2:** Deamination patterns observed in the two Šandalja samples, SA5 and SA6, after analysis with mapDamage.

## Data Availability

The mitochondrial consensus sequences and relevant genomic data generated in this study are publicly accessible at https://angen.agr.hr/en/318/Cubric‐Curik+et+al.+%282026.%29+Molecular+Ecology.
