# Supplementary material for: Genomic Diversity of Aurochs From a Mediterranean Ice‐Age Refugium
Source: Mol Ecol. 2026 Jun 29;35(13):e70449. doi: 10.1111/mec.70449 (PMC13315516; doi:10.1111/mec.70449)
Supplement: Supplementary file 2 — Figure S1: Radiocarbon date calibration curves of the two Šandalja samples. [file MEC-35-e70449-s002.pdf]

SA5

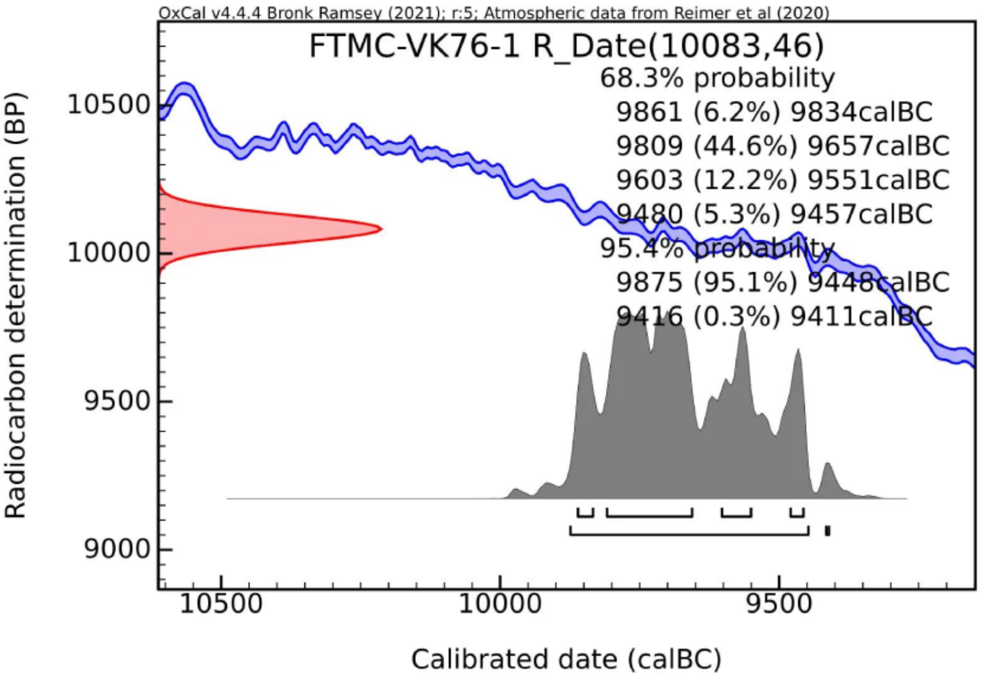

SA6

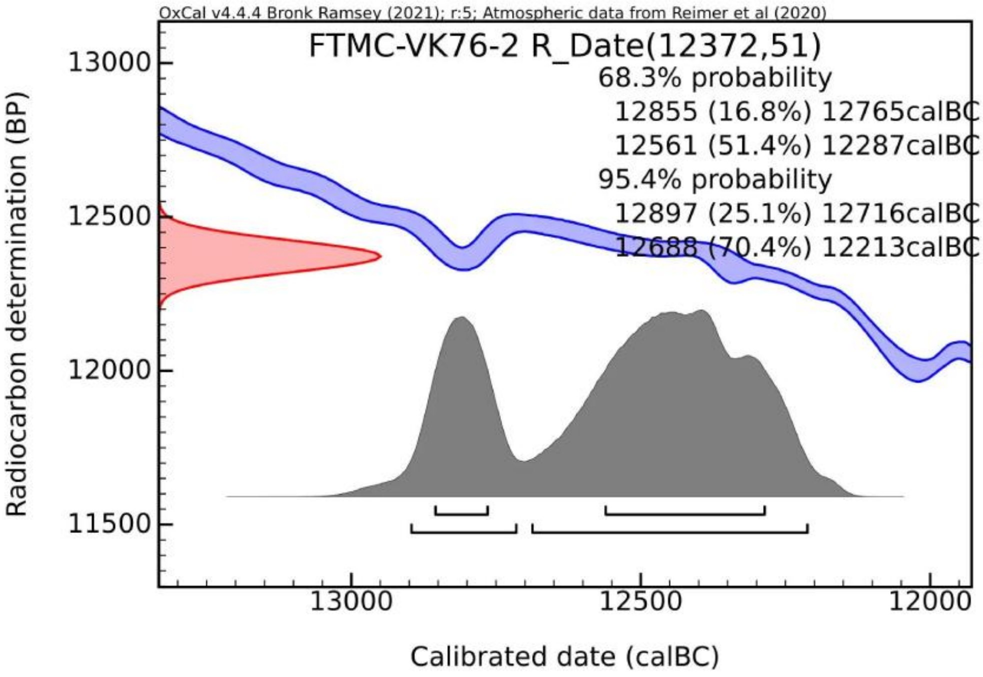

Figure S1. Radiocarbon dates calibration curves of the two Šandalja samples, SA5 and SA6, related to Table S1.
