# Supplementary material for: Genomic Diversity of Aurochs From a Mediterranean Ice‐Age Refugium
Source: Mol Ecol. 2026 Jun 29;35(13):e70449. doi: 10.1111/mec.70449 (PMC13315516; doi:10.1111/mec.70449)
Supplement: Supplementary file 3 — Figure S2: Deamination patterns observed in the two Šandalja samples, SA5 and SA6, after analysis with mapDamage. [file MEC-35-e70449-s003.pdf]

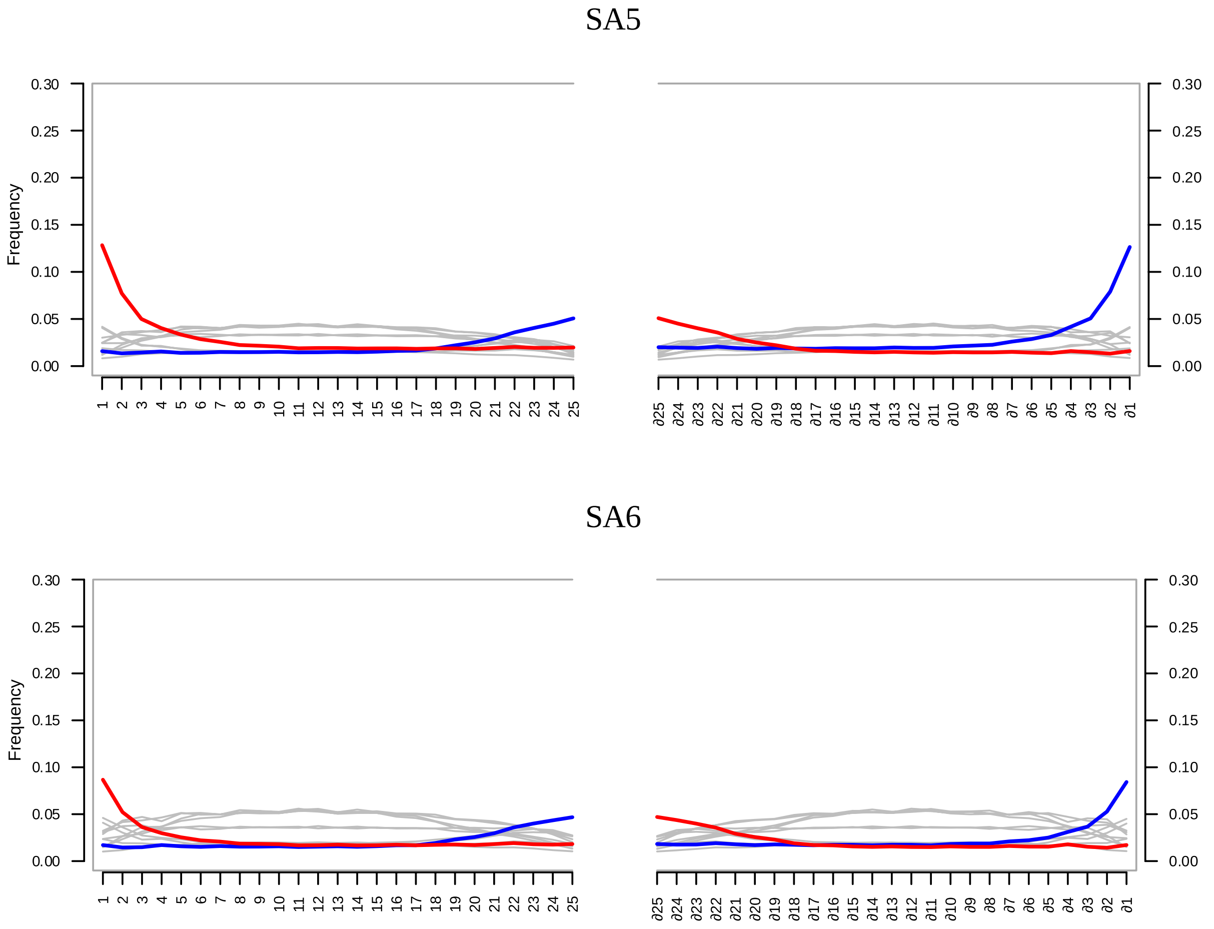

Figure S2. Deamination patterns observed in the two Šandalja samples, SA5 and SA6, after analysis with mapDamage, related to Table S1.
